# Experimental trauma rapidly modifies functional connectivity

**DOI:** 10.1007/s11682-020-00396-2

**Published:** 2020-09-28

**Authors:** Geraldine Gvozdanovic, Erich Seifritz, Philipp Stämpfli, Antonietta Canna, Björn Rasch, Fabrizio Esposito

**Affiliations:** 1 Department of Psychiatry, Psychotherapy and Psychosomatics, Psychiatric Hospital, University of Zurich, Zurich, Switzerland; 2 Institute of Psychology, University of Zurich, Zurich, Switzerland; 3 Zurich Center for Neuroeconomics, Department of Economics, University of Zurich, Zurich, Switzerland; 4 Competence Center of Sleep & Health Zurich, University of Zurich, Zurich, Switzerland; 5 Department of Medicine, Surgery and Dentistry “Scuola Medica Salernitana”, University of Salerno, Baronissi Salerno, Italy; 6 Department of Psychology, University of Fribourg, Fribourg, Switzerland

**Keywords:** Trauma, Resting state fMRI, Trauma film paradigm, Connectivity

## Abstract

Traumatic events can produce emotional, cognitive and autonomous physical responses. This may ultimately lead to post-traumatic stress disorder (PTSD), a psychiatric syndrome which requires comprehensive treatment. Trauma exposure alters functional connectivity; however, onset and nature of these changes are unknown. Here, we explore functional connectivity changes at rest directly after experimental trauma exposure. Seventy-three healthy subjects watched either a trauma or a control film. Resting state functional magnetic resonance imaging measurements were conducted before and directly after the film. Seed-based analyses revealed trauma-related changes in functional connectivity, specifically including decreases of connectivity between amygdala and middle temporal gyrus and increases between hippocampus and precuneus. These central effects were accompanied by trauma-related increases in heart rate. Moreover, connectivity between the amygdala and middle temporal gyrus predicted subsequent trauma-related valence. Our results demonstrate rapid functional connectivity changes in memory-related brain regions at rest after experimental trauma, selectively relating to changes in emotions evoked by the trauma manipulation. Results could represent an early predictive biomarker for the development of trauma-related PTSD and thus provide an indication for the need of early targeted preventive interventions.

**Electronic supplementary material:**

The online version of this article (10.1007/s11682-020-00396-2) contains supplementary material, which is available to authorized users.

## Introduction

Post-traumatic stress disorder (PTSD) is a psychiatric condition characterized by intrusive and avoidant symptoms, negative alterations in cognition and mood, and increases in arousal (APA 2013). Exposure to a traumatic event can alter neurobiological processing and ultimately result in PTSD (Bremner 1999; Elbert and Schauer 2014). Therefore, it is of great importance to explore early neurobiological factors following a traumatic event.

Previous research on resting state fMRI has been conducted mainly in full psychiatric manifestation of PTSD. These studies have a wide range of results, from showing that connectivity within the default mode network (DMN) has been found to be both enhanced (Lei et al. 2015) and suppressed (Bluhm et al. 2009; DiGangi et al. 2016; Sripada et al. 2012b; Tursich et al. 2015) in PTSD, while increased connectivity has been found in the salience network (SN) of PTSD patients (Nicholson et al. 2016; Sripada et al. 2012b). Connectivity within the DMN of PTSD patients has been reported to be associated with clinical measures on PTSD (Reuveni et al. 2016). Additional functional connectivity studies with amygdala seeds revealed an increased connectivity with the insula (Rabinak et al. 2011) and a decreased connectivity with the hippocampus and the anterior cingulate cortex (ACC) (Sripada et al. 2012a).

Only a small number of studies have looked at resting state functional connectivity (shortly) after trauma exposure and before full psychiatric manifestation of PTSD. These studies are of high relevance to identify possible early neurobiological factors involved in the prediction of PTSD symptoms. A previous study in rats revealed compromised intrinsic amygdala connectivity with the medial prefrontal cortex seven days after predator odor exposure (Liang et al. 2014). Patient data on resting state two days after trauma revealed altered connectivity of the posterior cingulate cortex (PCC), which is the main node of the DMN, with the right and left lingual gyrus, and right middle temporal gyrus (Qin et al. 2012). A further study focusing on the DMN within weeks after trauma exposure revealed an association of PCC with ACC, and amygdala resting-state connectivity with current symptoms (Lanius et al. 2010). In this case, amygdala connectivity predicted future PTSD development (Lanius et al. 2010). Interestingly, neural changes in the DMN and fronto-limbic striatal network within weeks after trauma exposure seemed to persist for over two years in the DMN, even when PTSD symptoms diminished (Du et al. 2015).

Heart rate measures at rest shortly after trauma revealed a lower heart rate that was of predictive value for the development of PTSD (Shaikh Al Arab et al. 2012). Further heart rate measures recorded during resting state fMRI analyses also revealed decreases in heart rate variability in PTSD; however, heart rate was not associated with PTSD-specific widespread central autonomic network connectivity (Thome et al. 2017). These results demonstrate the relevance of heart rate in PTSD. Interestingly, PTSD has been associated with higher heart rate responses in general (Liberzon and Abelson 2016; Morris et al. 2016; Pitman et al. 2012), resulting in the need to further evaluate the exact changes involved in heart rate responses after trauma exposure.

In sum, studies on resting state after trauma exposure indicate predictive neuroplastic changes within *days* to *weeks* after trauma exposure. However, these results did not report immediate neural changes within *minutes* after trauma. A disadvantage of measuring resting state days or weeks, instead of minutes, after trauma is that variables such as sleep, stress coping and other environmental factors may interact with PTSD development and intrusion symptoms (Holmes and Bourne 2008; Kleim et al. 2016; Meiser-Stedman et al. 2009; Woud et al. 2012). Particularly, these factors could potentially alter the original structure of trauma. Thus, the possibility of drawing a strong conclusion about the exact nature of trauma and its influence on brain function and structures after days or weeks remains limited.

The present study is the first to use both pre- and post-resting state measurements, the latter immediately after experimental trauma. To this extent, the validated and well established trauma film paradigm is applied in healthy subjects (Holmes and Bourne 2008). As one of the seed regions, we selected the amygdala, which is associated with threat (Dolan and Vuilleumier 2003; Lang et al. 2000; LeDoux 2003) and is of central importance in PTSD (Pitman et al. 2012). We hypothesize altered functional connectivity between the amygdala and emotion/memory related brain regions for the trauma film group, measured at rest after trauma film exposure, compared to pre-film resting state and compared to the resting state scans of the control film group. As a further seed region of interest the hippocampus, which is associated with episodic memory, was selected. (Brodt et al. 2016; Frankland and Bontempi 2005; Stickgold 2002). We hypothesize that the hippocampus will be associated with altered memory integration into the neocortex for the trauma film group after the film compared to pre-film resting state and compared to resting state scans of the control film group. Thus, these selected seed regions are expected to be of central importance for threat and memory processing after experimental trauma.

## Methods

### Participants

Eighty healthy, non-smoking, right-handed female participants underwent a general screening to exclude present and past medical and psychiatric disorders. Previous exposure to a traumatic event was also an exclusion criterion. Participants were screened for standard MRI exclusion criteria. Due to movement during scans, seven participants had to be discarded from the experiment, leaving seventy-three healthy participants in the sample (mean age 23.4, range 18–36 years). Subjects provided their written informed consent after the study protocol had been fully explained. Additionally, subjects were reimbursed for their participation (CHF 25/h). The study protocol was in line with the Declaration of Helsinki and was approved by a local ethical review board.

### Experimental procedure

Participants arrived at the imaging center and gave their informed consent. At first, they were informed about the experimental procedure. After entering the scanner, half of the subjects (*n* = 37) watched a trauma film (experimental trauma group) and the other half (*n* = 36) watched a neutral film (control group), in accordance with a between-subject design. Resting state fMRI-measurements were conducted before (pre-resting state) and after the film (post-resting state) in both the experimental trauma and the control group (for an overview, please see Fig. 1 of the Supplementary Material).

**Fig. 1:**
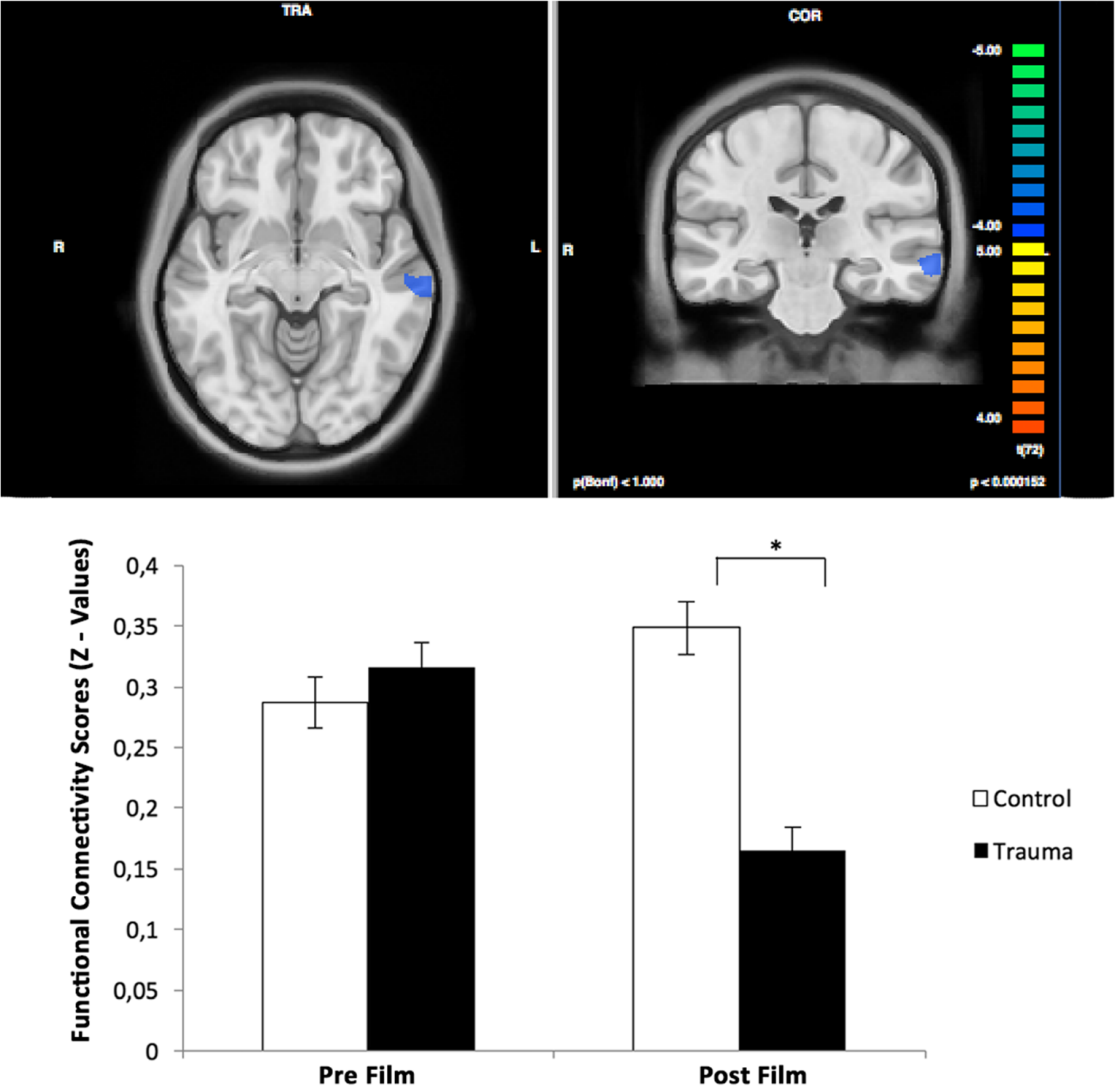
Functional connectivity analyses with left amygdala seed resulted into a decreased connectivity to the left middle temporal gyrus

Participants were instructed to lay still, close their eyes, think of nothing specific and not fall asleep during resting state fMRI measurements. Each session lasted for six minutes, with one resting state directly before the film and one directly after the film.

In parallel to the resting state fMRI measurements, heart rate (electrocardiography, ECG) measurements were also recorded.

After fMRI measurements, participants rated pictures from the previously seen film together with international affective pictures system (IAPS) and scrambled pictures (Lang et al. 2008). Additionally, they received an intrusion diary with a detailed explanation for the following seven days. Both the trauma film group and the control film group filled in the trauma diary.

### Film paradigm

The trauma film paradigm was first developed in the 60s (Horowitz 1969; Lazarus 1964) and it has been successfully applied in over 70 studies since 2008 (James et al. 2016). By definition, the trauma film paradigm is an experimental medicine model. Experimental medicine models try to model deviant mechanisms in non-patient samples in order to evaluate processes involved in the development of clinically relevant symptoms (James et al. 2016). In this case, the experimental medicine model evaluates functional connectivity in non-patient groups in order to study mechanisms potentially leading to PTSD (James et al. 2016).

Responses comparable to real-life trauma have been systematically observed in healthy subjects after an experimental trauma (i.e. trauma film) (James et al. 2016). These include hallmark symptoms of PTSD, such as physiological arousal, intrusive memories, negative cognitions and mood have been reported (APA 2013; Butler et al. 1995; Holmes et al. 2004; James et al. 2016; Weidmann et al. 2009). However, unlike in PTSD, symptoms that arise after an experimental trauma only last for hours or days and decline within one week (Holmes et al. 2004; James et al. 2016). Thus, the trauma film paradigm provides an optimal setting to bridge non-clinical and clinical samples in a highly controlled setting in order to examine the development of PTSD symptoms (Clark and Mackay 2015).

The traumatic film used in this study consisted of a rape scene that resembles witnessing a traumatic event. According to the diagnosis criteria for PTSD, both direct exposure to a traumatic event and witnessing trauma can result in PTSD (APA 2013). Previous research on the trauma film paradigm revealed that when comparing different trauma films, our selected trauma film with a rape scene had the highest physiological, emotional and intrusive memory impact on participants, in both females and males. Nevertheless, we expect stronger effects in female subjects due to a higher relatability to the main character in the rape scene, a young female heading back from a party alone at night and being raped by a stranger.

In accordance with the trauma film paradigm (Holmes and Bourne 2008) participants either watched a trauma or a control film (duration of both film clips: 14 min). In the trauma film, rape and further physical assault was depicted (film called “Irreversible”, Noe and Rossignon 2002). In the neutral film, a collection of neutral scenes with the same characters was displayed. Both films had the same duration. Participants had to rate their current mental state (MDBF, (Steyer et al. 1997)) before and after the film.

### Emotional picture ratings

Participants had to rate emotional pictures of different categories according to valence and arousal on an 11-point Likert scale (ranging from 0 “neutral” to 10 “very negative”) 2 h after film exposure. Categories included 20 film pictures, 20 negative and 20 neutral pictures taken from the international affective pictures system (IAPS) (Lang et al. 2008) and 20 scrambled pictures. Both neutral and negative IAPS pictures were matched in brightness to the film pictures. Scrambled pictures consisted of a collage of all pictures and were blurred with a Gaussian filter. Notably, our film pictures consisted of emotionally neutral pictures taken from both films equally to prevent familiarity effects (trauma and neutral films). They included no action but rather served as a (initially neutral) reminder cue (e.g. tunnel where the protagonist was raped). Thus, potential differences between the groups in ratings of the trauma pictures can only be related to effects generated by the experimental trauma itself.

### Questionnaires

Before and after the film, participants rated their current mental state on a standardized scale (“Mehrdimensionale Befindlichkeitsfragebogen” MDBF (Steyer et al. 1997)). The MDBF is a well established German questionnaire on current mental state. It has an internal consistency (Chronbachs Alpha) between α = .73 and α = .89 or a reliability between .87 und .97 (Steyer et al. 1997). The scale consists of three dimensions: good – bad mood, alertness – tiredness and calmness – restlessness. We have used the 12 item MDBF short version “A”. Each item had a 5-point Likert scale ranging from not at all (1) to very (5).

We have performed a repeated measured ANOVA with the MDBF score (pre-film vs post-film) as a within-subjects’ factor and film group (trauma vs control) as a between-subjects’ factor. We thereby aimed at demonstrating a potential effectiveness of the trauma film paradigm.

### Intrusion diary

The intrusion diary consisted of a seven-day diary that had to be filled in at home. Participants were instructed about intrusive memories and asked to write down every intrusive memory that came to their mind. This included content, vividness, distress, and intrusive characteristics for every memory that was experienced daily during this time. Intrusion diaries were analyzed and the sum of reported intrusions per week was used for further analyses. Scoring criteria for intrusions included ratings on distress, vividness, and sudden occurrences of the reported memories. In total two psychologists scored the diaries, with a third psychologist reviewing the decision in case of clear divisiveness. There was an inter-rater reliability of r = .86 between the reviewers of the diary.

### Heart rate data acquisition and analyses

Heart rate monitoring was performed with an electrocardiography (ECG) system used during fMRI (Philips Healthcare, Best, The Netherlands). Changes in heart rate were monitored with a four-electrode system at a sampling rate of 500 Hz. Physiological data were analyzed with the physio toolbox Translational Algorithms for Psychiatry-Advancing Science (TAPAS; http://www.translationalneuromodeling.org/tapas/) and in-house scripts using MATLAB R2016b (The Mathwords Inc., Natick, USA). For heart rate data, the number of heart beats and heart rate variability using R-R intervals were computed for the entire 6 min of rest. Correction for physiological noise was performed via RETROICOR (Glover et al. 2000; Hutton et al. 2011) using Fourier expansions of different order for the estimated phases of cardiac pulsation (3rd order), respiration (4th order) and cardiorespiratory interactions (1st order) (Hutton et al. 2011). The corresponding beats per minute as well as heart rate variability were calculated with the Matlab PhysIO Toolbox (Kasper et al. 2009) (algorithm for heart beats detection open source code available as part of the TAPAS software collection). Due to technical problems during heart rate recordings, seven participants had to be discarded from the sample.

For further analyses we have conducted a repeated measures ANOVA with mean heart rate during scan (6 min pre-film resting state and 6 min post-film resting state) as a within- subjects’ factor and film group (trauma vs control) as a between-subjects factor.

### Functional MRI data acquisition

Measurements were performed on a Philips Achieva 3.0 Tesla TX whole-body magnetic resonance scanner (Philips Healthcare, Best, The Netherlands), equipped with a thirty-two channel head coil array. Functional images were collected using a sensitivity-encoded single-shot echo-planar (EPI) sequence (repetition time TR = 2000 ms; echo time TE = 30 ms; field of view = 240 × 240 mm^2^; in-plane resolution = 3 × 3 mm^2^; slice thickness = 4 mm; 35 axial slices; no slice gap; sensitivity-encoded acceleration factor (SENSE) = 2.0) sensitive to BOLD contrast (T2* weighting). Using a midsagittal scout image, the slices were placed along the anterior–posterior commissure plane covering the entire brain. Additionally, a 3D T1-weighted anatomical scan was obtained for structural reference (sagittal; field of view = 240 × 240 mm^2^, slices = 160; in-plane voxel size = 1 × 1 mm^2^; flip angle = 8 °; TR = 9.3 ms; TE = 3.7 ms). All tasks and films were presented on MR-compatible video goggles (Resonance Technology, Northridge, USA). Furthermore, for good sound quality, headphones especially designed for MR measurements (MR confon GmbH, Magdeburg, Germany) were used.

### Image analysis

Image data processing was performed in Matlab R2016b (The Mathwords Inc., Natick, USA) and BrainVoyager QX (Brain Innovation B. V., Maastricht, The Netherlands).

Standard image data preparation and preprocessing was performed with the Matlab toolbox DPABI (http://rfmri.org/dpabi) which is based on SPM12 functions (www.fil.ion.ucl.ac.uk/spm). Functional data preprocessing included a correction for slice scan timing acquisition, realignment to the first volume using rigid body transformation, normalization to the EPI template in Montreal Neurological Institute (MNI) space, reslicing to voxel resolution of 3 × 3 × 3 mm^3^ and spatial smoothing using a 6 mm full width at half maximum Gaussian kernel. To regress out any residual effects of motion (micro-motion) from each data set prior to seed-based functional connectivity analyses, the so-called “Friston-24” model was used. This model incorporates the six head motion translation and rotation parameters estimated during 3D rigid body correction, their first order derivatives and all corresponding squared items (Friston et al. 1996; Satterthwaite et al. 2013). In addition, nuisance physiological signals from white matter (WM) and cerebro-spinal fluid (CSF) template masks in MNI space were also extracted, thereby following previous recommendations (Geerligs et al. 2017; Shirer et al. 2015; Varikuti et al. 2017), all 24 motion parameters, together with the WM and CSF signals, were regressed out from each time-course at each voxel. The data was band-pass filtered between 0.01 and 0.1 Hz after nuisance regression.

Mean framewise displacement (FD) was also calculated to quantify head micro-movements between consecutive pairs of brain volumes (Power et al. 2014). Namely, for each scan, mean FD was also obtained from the sum of absolute values of differentiated movement estimates per time-point divided by the number of time- points (Power et al. 2012). The realignment threshold for the mean FD applied to our data was: FD_mean_ and FD_max_ < 0.5 mm, according to previous research (Power et al. 2012).

To rule out the possibility that the observed effects were spurious effects mediated by head motion, we fitted a number of ANOVA models on our motion estimates to assess the significance of any factor or variable in terms of the influence of motion. No effect of motion was found. A detailed description of the motion correction results can be found in the Supplementary Materials.

For seed-based functional connectivity analyses, amygdala and hippocampus/parahippocampal gyrus seeds were predefined based on coordinates from meta-analyses. The meta-analyses of Koch et al. (2016) and Wang et al. (2016) on resting state connectivity provided MNI [x y z] coordinates of −28, 0, −26 for the amygdala (Wang et al. 2016) and MNI coordinates of −32 − 10 − 18 for the hippocampus/parahippocampal gyrus (Koch et al. 2016). As the reported MNI coordinates from the meta analyses were unilateral, we defined amygdala and hippocampus seeds for both hemispheres. Specifically, the coordinates of a contra-lateral seed were obtained by mirroring the coordinates of the reported unilateral seed, i.e. by inverting the sign of the X coordinate while keeping the Y and Z coordinates identical. In this way, the seed-based functional connectivity analyses were separately performed for both the left and the right seed (unilateral seed analyses).

The seed-based functional connectivity analysis was performed in BrainVoyager QX. For further use in BrainVoyager QX, MNI- coordinates of the seeds were transformed into Talairach-coordinates. A sphere with a radius of 4 mm was drawn around the coordinates in BrainVoyager. Anatomical and functional data sets were imported from the initial NIFTI format and MNI space into BrainVoyager format and transformed to Talairach space using the Matlab toolbox Neuroelf (http://neuroelf.org) and the Matlab COM interface of BrainVoyager QX. Time courses of the predefined regions amygdala and hippocampus/parahippocampal gyrus were extracted and averaged from each individual. To compute functional connectivity maps, the mean regional time course was correlated against the time course of each voxel of the brain. Thereby separate correlation maps were produced for each subject, scan and ROI. The correlation maps were applied the Fisher’s r-to-z transform z = 0.5 Ln [(1 + r) / (1 − r)] before entering the second-level random-effects statistical analysis. For this analysis, the functional connectivity z-maps from all subjects and scans were combined and entered into the analysis of (co)variance (ANOVA) module of BrainVoyager QX. Here, a 2-way mixed-effects ANOVA design was specified with one within-subject factor (“scan “) and one between-subject factor (“trauma / film type”). Following the experimental design, the factor “scan” was assigned with two levels (pre-film, post-film), the factor “trauma / film type” with two levels (experimental trauma group, control group). After implementing the general linear model, to detect any effects of systematic functional connectivity changes related to film and trauma, a linear contrast was calculated for the post-film scan in the experimental trauma group versus the pre-film scan in the experimental trauma group and the pre- and post-film scans in the control group. We performed a targeted contrast from the overall ANOVA model in which the numbers were set to balance the weights across the four conditions. The t-statistics for the resulting contrast (+3 post-film trauma, −1 pre-film trauma, −1 post-film-control, −1 pre-film control) was thus computed at each voxel, yielding a whole-brain t-map which was overlaid in pseudo-color onto the MNI template. To protect against false positives and correct for multiple comparisons, only statistically significant regional effects were displayed for compact clusters surviving the joint application of a voxel- and a cluster-level threshold, which were chosen using a non-parametric randomization approach based on Monte Carlo simulations. Namely, a strict cluster-forming (uncorrected) threshold (*p* = 0.0005) was initially applied to all voxels (Eklund et al. 2016); then, the thresholded maps were submitted to a whole-brain correction criterion which was based on the estimate of the maps’ spatial smoothness using the 3-d extension of the formula in (Forman et al. 1995) and on an iterative procedure for estimating cluster-level false-positive rates (Monte Carlo simulation). To match the level of “smoothness” between the calculated and the simulated images, the contrast images were used to estimate the spatial smoothness of the data used for correction. To calculate this, a BrainVoyager QX plugin was used to estimate the FWHM of the Gaussian kernel appearing in the 3D extension of the formula reported in (Forman et al. 1995). A minimum cluster size was set in such a way that an average of 5/N % false positive clusters were counted in 1000 randomly generated images to which the same thresholds were applied, N being an additional correction for the number of seeds that was applied using the Bonferroni criterion. According to the Monte Carlo simulations, at the used cluster defining threshold of *p* = 0.0005, the minimum cluster size ranged from 189 mm3 to 270 mm3 depending on the estimated smoothness. These criteria were applied to all our image analyses.

### Brain behavior correlates

For regions identified in the above analysis, mean regional functional connectivity z-values were extracted for post-film scans per subject for both the experimental trauma group and the control group. These extracted connectivity values were then used for regression and correlational analyses, respectively, with behavioral data (ratings on film pictures concerning valence and arousal, diary intrusions) and heart rate measurements.

We applied linear regression models for behavioral data with film picture ratings and intrusions as dependent variables. Therefore, our connectivity data had the potential to serve as predictors (independent variables). Our main interest was to look for associations between trauma specific behavior and trauma related neural correlates, thus we conducted these analyses only for the trauma group.

As heart rate measurements were recorded simultaneously during resting state fMRI in both groups, correlational analyses between functional connectivity and heart rate data were conducted for both the trauma and the control group.

In both analyses, only post-film scans were considered, as these corresponded to the actual timepoint where trauma specific neural effects would eventually become manifest in a real-world scenario (where no pre-film scans would be available).

For the extraction of functional connectivity z-values, the clusters resulting from the targeted contrast of interest in the seed-based analyses were considered. This was done by drawing a 4 mm sphere around the cluster peaks. Then, we performed three linear regression analyses using the extracted z-values and the behavioral data as predictor (independent) and outcome (dependent) variables respectively. Namely, for each film picture rating (arousal and valence) and for intrusions, a separate regression analysis was performed where the connectivity z-values from both clusters served as independent variables.

Due to outlier detection, two participants had to be excluded from the sample.

## Results

### Effectiveness of the trauma film paradigm

#### Current mental state

Results revealed a significant main effect of current mental state score (pre-film vs post-film) *F*_1,71_ = 99.40, *p* < 0.001. Moreover, there was a significant current mental state score (pre-film vs post-film) x film group (trauma vs control group) interaction effect *F*_1,71_ = 63.04, *p* < 0.001. Post-hoc t-tests revealed a decrease in current mental state score (more negative current state) after the film compared to before the film for both the trauma film group (*t*_36_ = 9.89, *p* < 0.001; MDBF score pre-film: μ_pre =_ 48.19; post-film μ_post_ = 33.97) and the control film group (*t*_35_ = 2.53, *p* < 0.05; MDBF score prefilm: μ_pre =_ 48.94; post-film μ_post_ = 47.33). Moreover, there was a significant difference between the trauma film group and the control film only for post-film current mental state score (*t*_63.74_ = 7.88, *p* < 0.001).

#### Emotional picture ratings

Results revealed a significant main effect for picture type (*F*_3,207_ = 280.1, *p* < 0.001) and a significant interaction effect of picture type (film pictures, negative IAPS pictures, neutral pictures and scrambled) x film group (trauma film and control film) for both valence ratings (*F*_3,207_ = 17.2, *p* < 0.001) and arousal ratings (*F*_3,207_ = 13.9, *p* < 0.001). Post-hoc analyses revealed a significant difference between the trauma and control group only for film pictures ratings in valence (*t*_70_ = − 4.9, *p* < 0.001) and arousal (*t*_70_ = −5.1, *p* < 0.001). For an overview of descriptive data on the emotional picture ratings, please refer to the Supplementary Material.

#### Intrusions

In order to further evaluate the effectiveness of the paradigm, we tested whether there were differences in the number of intrusions between the experimental trauma and the control group. Due to missing diaries in the control group, 4 participants had to be excluded from the sample. There was a significant difference in the number of intrusions between the experimental trauma film group and the control film group (*F*_68 =_ 23.225, *p* < 0.001). For descriptive data please refer to the Supplementary Materials.

#### Heart rate data

Participants’ heart rate was analyzed independently of brain data. Heart rate was measured during resting state fMRI, thus while participants were at rest. Heart rate was measured both before and after the film (timepoint) for both the trauma and the control film group. Results of our repeated measures ANOVA on heart rate data revealed a significant main effect of timepoint (heart rate before the film vs heart rate after the film), F _*1,64*_ = 8.97, *p* < 0.01. Moreover, we have found a marginally significant timepoint (heart rate before the film vs heart rate after the film) x film group (trauma vs control) interaction effect, F _*1,64*_ = 3.12, *p* = 0.08. Post-hoc tests reveal that only within the trauma film group there was a significantly higher number of heart beats after the film compared to before the film, t_*(34)*_ = − 3.92, *p* < 0.001 .

We further examined whether there was a significant correlation between heart rate and post-film amygdala-MTG and hippocampus -precuneus connectivity, within both control and experimental trauma participant subsamples. HR data did not correlate with post-film functional connectivity z-values in none of the two groups.

#### Amygdala seeds

For the targeted contrast, functional connectivity between the amygdala seeds and all voxels from the whole brain revealed a decreased connectivity between the left amygdala seed (Talairach [x y z] = [−28, −3, −19]) and left middle temporal gyrus (MTG) (*F*_*peak* =_ − 4.69; Talairach [x y z] = [−63, −19, −8]; cluster size = 924 mm3) for the post-film resting state trauma compared to all other control conditions (pre-film resting state trauma, pre-film resting state control and post-film resting state control) (Fig. 1). We found no significant results for the right amygdala seed.

ANOVA results revealed a film type x scan interaction effect for the left and right amygdala seed that mainly affected temporal - and frontal gyri. For detailed ANOVA results using the amygdala seeds please refer to the Supplementary Materials.

#### Hippocampus/parahippocampal gyrus seeds

**Fig. 2:**
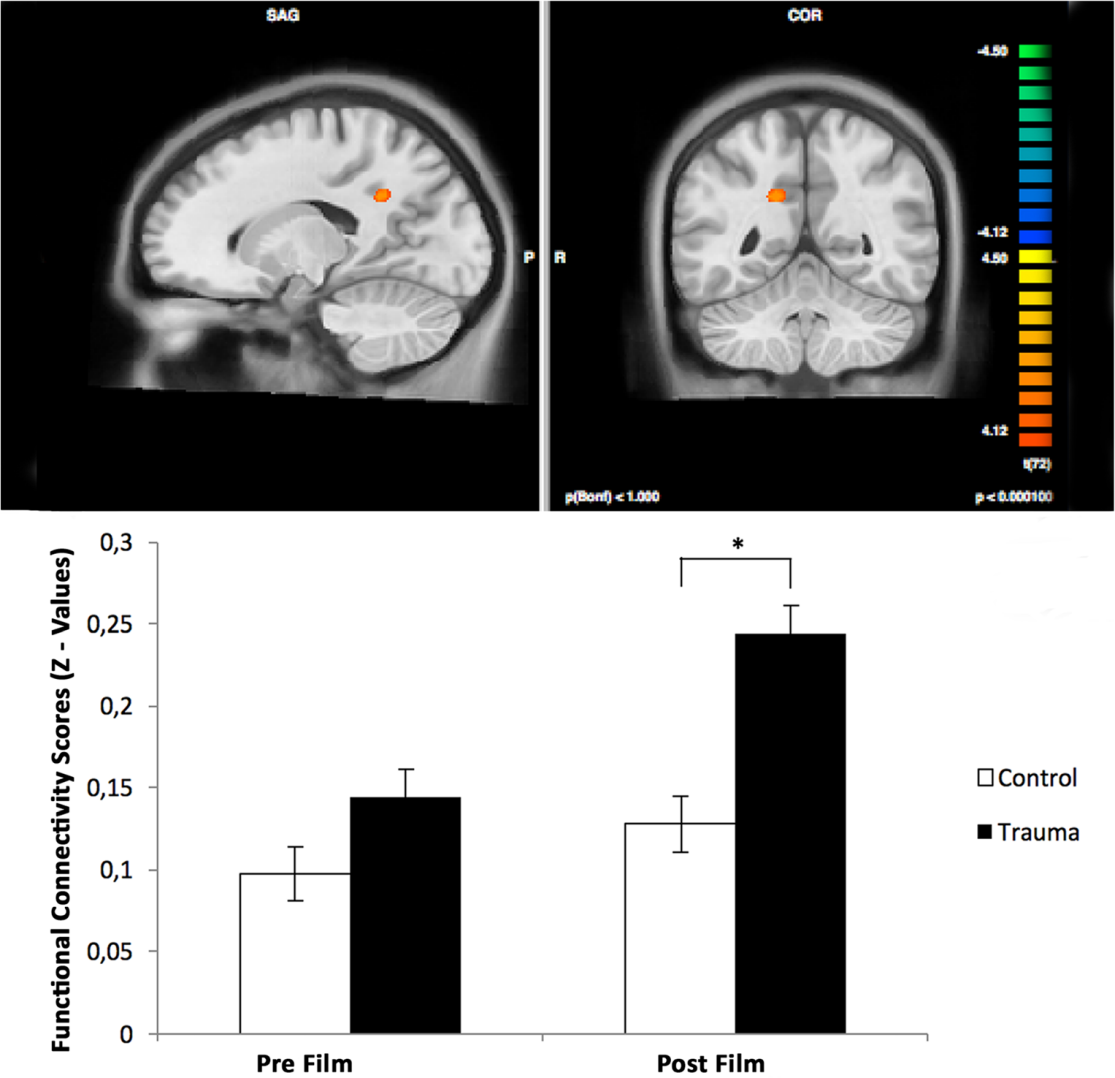
Functional connectivity analyses with right hippocampus/parahippocampal gyrus seed resulted in an increased connectivity to the right precuneus

For the targeted contrast, the hippocampus seeds revealed an increased functional connectivity of the right hippocampus seed (Talairach [x y z] = [30–12 − 11]) with the right precuneus (*F*_*peak* =_ 4.74; Talairach [x y z] = [15, −46, 31]; cluster size = 1090 mm3) for the post-film resting state of the experimental trauma group compared to all other resting states, i.e. the pre-film resting state connectivity of both groups and the post-film resting state connectivity of the control group (Fig. 2).

In addition, no difference was found between the film groups in pre-scan for the amygdala and also for the hippocampus ROIs (post-hoc t-test: *t* (71) = .49, *p =* .62) (Fig. 2).

We found no significant results for the left hippocampus seed.

ANOVA results revealed a significant main effect of scan for the right hippocampus seed and the left precuneus. Moreover, there was a main effect for film type in the medial frontal gyrus. For detailed ANOVA results using the hippocampus seeds please refer to the Supplementary Materials.

#### Brain behavior correlates

In our linear regression analyses, ratings on film pictures (delta values to scrambled pictures) and intrusions were entered into the model as dependent variables. In total we ran three linear regression analyses: In the first, valence ratings of film pictures served as outcome (dependent) variable. In the second, arousal ratings of film pictures served as outcome (dependent) variable. In the third model, intrusions served as outcome (dependent) variable. For all three models, extracted z-values of the MTG and the precuneus (i.e. the two clusters that survived multiple comparison corrections on group level in the seed-based analyses using amygdala and hippocampus seeds) were entered as independent variables into the model. These analyses were done for subjects in the trauma group after film exposure.

**Fig. 3:**
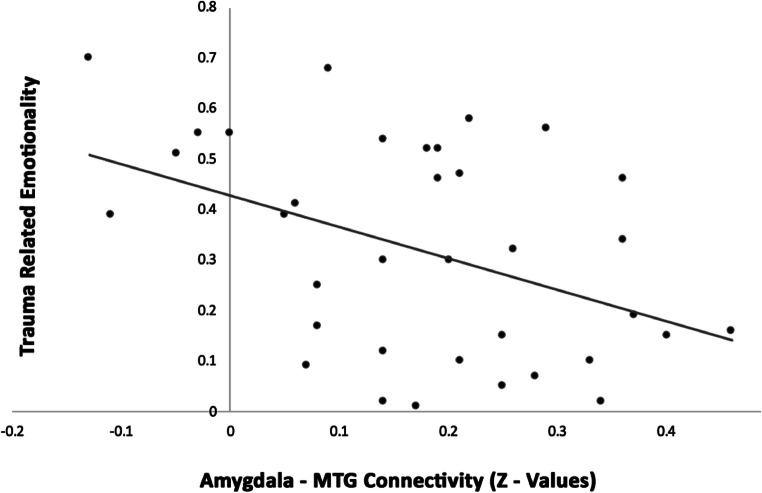
Brain-behavior correlates revealing a predictive role of the amygdala – middle temporal gyrus connectivity and subsequent trauma related valence

In the first model (adjusted R^2^ = 0.147, F[2,32] = 3.967, *p* = 0.029), the amygdala - MTG connectivity was revealed to have a significant negative influence on the valence ratings of film pictures (t[32] = −2.249, *p* = 0.032), while the hippocampus - precuneus connectivity was not of significant influence within the model (t[32] = −0.795, *p* = 0.432). Thus, brain behavior analyses revealed a significant prediction of the amygdala - MTG connectivity and subsequent valence ratings, see Fig. 3. Neither the second nor the third model reached the statistical significance.

Descriptive data on the intrusion diary can be found in the Supplementary Material.

## Discussion

Our study delineates resting-state functional connectivity changes immediately after experimental trauma using amygdala and hippocampus seeds.

Functional images were acquired during six minutes before and immediately after a trauma film to prevent interactions with environmental factors.

The effectiveness of the paradigm was validated by significant changes in current mental state, trauma related valence and arousal, intrusions and heart rate induced by the experimental trauma condition. We found a decrease in connectivity between the amygdala and the MTG, and an increase in connectivity between the hippocampus and precuneus in the trauma film group compared to the control film group. The functional connectivity between the amygdala and the MTG predicted trauma related valence.

Changes in heart rate validated the effectiveness of the trauma film paradigm by revealing that the trauma film group displayed significantly higher heart rate responses immediately after the film compared to the period preceding the film. This finding is in line with previous research. A meta-analysis of physiological measurements (Morris et al. 2016) emphasizes the importance of altered heart rate signals for PTSD development shortly after trauma exposure. More precisely, a significant correlation was reported between higher heart rate within hours after trauma exposure and higher PTSD symptoms at a follow-up screening (Morris et al. 2016).

In view of the established high relevance of the amygdala during processing of threat and anxiety at early stages (Dolan and Vuilleumier 2003; P. J. Lang et al. 2000; LeDoux 2003), our first functional connectivity analyses focused on amygdala seeds. We found a decreased functional connectivity between the amygdala and the MTG. Our result of connectivity changes in the amygdala - MTG partly overlaps with previous resting state fMRI studies in PTSD, albeit from seeds placed in different brain structures (Kennis et al. 2015; Qin et al. 2012). Specifically, decreases in functional connectivity between a perigenual ACC seed, the superior medial gyrus, and MTG were found in veterans with and without PTSD compared to healthy controls (Kennis et al. 2015). When looking at a PCC seed, PTSD was associated with altered functional connectivity amongst others in the right and left MTG compared to a control group (Qin et al. 2012).

Additionally, further research on the neural activity during encoding of trauma film scenes revealed the relevance of MTG for actually intrusive scenes compared to potentially intrusive scenes (Clark et al. 2016). This indicates that the amygdala - MTG connectivity might be specifically involved in memory processing of intrusive negative contents of the film during rest.

The amygdala also seems to play an essential role in memory formation and consolidation. Specifically, the amygdala seems to have an important function in modulating emotional and arousing episodic memory processing in both an immediate and a sustained fashion (Gagnon and Wagner 2016). After exposure to an episodic event, emotional arousal first enhances episodic memory processing by activating the noradrenergic basolateral amygdala (Gagnon and Wagner 2016). Subsequently, the amygdala modulates encoding and consolidation processes in the medial temporal lobe. (Gagnon and Wagner 2016; McGaugh et al. 1996). It is hypothesized that threat captures attention, creates arousal and enhances memory, ultimately resulting in rapid catecholamine responses (such as noradrenaline) (Gagnon and Wagner 2016). The release of noradrenaline additionally enhances memory consolidation processes (Gagnon and Wagner 2016). Thus, a decrease in functional connectivity emerging from an amygdala seed during rest might signal an early relevant modulation of memory processing after a traumatic event. Nevertheless, the authors did not specifically mention the role of other temporal lobe regions (such as the MTG) that might be of relevance in memory formation together with the amygdala. Therefore, it still remains to be clarified in future research how interactions of the amygdala and the MTG specifically emerge during memory formation.

Interestingly, a possible mechanistic nature of amygdala functional connectivity changes in trauma related memory processing is suggested by our observed brain behavior correlates. The extracted regional MTG scores (derived from connectivity with amygdala seeds) were significantly predictive of subsequent trauma related valence, with lower connectivity in the amygdala - MTG being associated with higher perceived emotionality. This indicates that, potentially due to a lack of enhancement or an inadequate modulation by the amygdala, a maladaptive memory integration takes place which ultimately predicts higher emotionality associated with the trauma memory. This hints in the direction of a rapid role of the amygdala - MTG in memory formation, influencing subsequent emotional perception of trauma reminders within hours after exposure. Recent research has also discussed PTSD as a temporal lobe disorder, which might be linked to the reexperience component of PTSD (Engdahl et al. 2010). This theory is consistent with our interaction effects of scan and film type using amygdala seeds that mainly affected temporal gyri and frontal gyri. Frontal lobe dysfunction has also been associated with core symptomatology in PTSD (Selemon et al. 2019).

Our results are in line with other previous research on the promising effects of predictive values of early neural correlates for symptom development using a trauma film paradigm (Clark et al. 2014). Specifically, we were also able to demonstrate that connectivity changes after experimental trauma exposure emerge early (while using the same paradigm) and that these changes have a predictive potential for trauma related valence.

As for our connectivity analyses using a hippocampus seed, our result on altered hippocampal functioning and connectivity is consistent with recent studies that identified the hippocampus as a central region involved in PTSD development, particularly due to its role in context conditioning (Liberzon and Abelson 2016). When responding to partial cues, the malfunctioning of the hippocampus has been shown to lead to generalized fear responses that are incongruous with the current context (Liberzon and Abelson 2016).

Indeed, a previous meta-analysis of trauma-memory processing revealed that the precuneus is strongly activated during trauma-related stimuli (Sartory et al. 2013). Thus, our result on functional connectivity enhancement between the precuneus and hippocampus by a recent trauma film experience is consistent with this function of the precuneus. Interestingly, the precuneus also seems to have a prominent role in episodic memory formation (Cabeza et al. 2008; Cavanna and Trimble 2006), a function that has been previously shown as altered in PTSD (Crespo and Fernandez-Lansac 2016).

It is worth noting that a recent paper by Brodt et al. (Brodt et al. 2016) also pointed out the joint relevance of the hippocampus and precuneus in rapid memory formation. In fact, the precuneus activity was positively correlated with rapidly changing memory performance. Although these findings contradict prior literature on the slow nature of memory formation processes (taking days up to months), they are consistent with our results of a stronger connectivity between the hippocampus and the precuneus observed in resting state immediately after trauma film exposure.

The results of Brodt et al. (Brodt et al. 2016) might also hint in the direction of a stronger memory trace emerging after trauma, potentially leading to an overrepresentation of the trauma memory. However, we did not find any correlations between the hippocampus - precuneus connectivity and the incidence of intrusive memories during the subsequent seven days. Therefore, the exact nature of our increased connectivity between the right hippocampus and the right precuneus and its precise influence on symptom development remains unclear.

Moreover, it also remains to be investigated in what way the precuneus plays a role in episodic memory formation independent of the traumatic content itself. This is indicated by our main effect of scan between the right hippocampus and left precuneus, revealing a potentially rather general effect of episodic memory consolidation of the presented films.

Further, our main effect of experimental trauma (film type, see Supplementary Material) on the connectivity between the hippocampus and the vmPFC/medial frontal gyrus is in line with previous research on memory formation, where the connectivity between these regions seems to be associated with highly emotional arousing autobiographical memories (Nawa and Ando 2019) and different aspects of episodic memory (Eichenbaum 2017).

As a consequence of our results, an immediate treatment might be of great importance to prevent PTSD symptom development. Glucocorticoids might be considered as a potential means for immediate treatment. Due to their general role in memory consolidation processes, glucocorticoids have been indicated as a potentially useful early preventive treatment (Quervain et al. 2017). Emotional memories are initially consolidated within hours (McGaugh 2000), thus a time frame window for interventions is provided (James et al. 2015). Interestingly, PTSD has been associated with low cortisol levels (cortisol is a subcategory of glucocorticoids), although stress normally leads to high cortisol levels (Quervain et al. 2017; Liberzon and Abelson 2016; Pitman et al. 2012). This cortisol paradox of reported low levels in PTSD despite subjective reports of high stress remains not fully understood. However, our results reveal that memory-related connectivity is altered immediately after experimental trauma. This gives new indications for a potential usefulness of early and targeted application of glucocorticoids (Quervain et al. 2007; Quervain et al. 2017). Indeed, a preliminary study reveals that the application of a high dosage of cortisol within hours after trauma exposure results in a significant reduction of later PTSD development (Zohar et al. 2011).

Taken together, our findings give a new perspective on the nature of functional connectivity changes due to trauma exposure in an experimental setting while avoiding the interference of environmental factors (e.g. sleep, stress coping). Recent literature supports the potential relevance of our findings by stating that trauma exposure, rather than PTSD itself, is related to changes in functional connectivity (DiGangi et al. 2016). Moreover, another study indicates that trauma characteristics including time after trauma (i.e. number of years) modify functional connectivity in the right parietal region of the DMN in PTSD patients and trauma controls (Reuveni et al. 2016). In our results, experimental trauma exposure modifies functional connectivity already at the earliest stages. The necessity to employ interventions in traumatized patients at the earliest time-point after trauma exposure should be considered in relation to the design and the targets of post-trauma interventions. In fact, our results clearly suggest that rapid changes in functional connectivity may already be rapidly occurring after exposure to a single trauma event. Interestingly, previous studies revealed that exposure to multiple traumas results in a higher prevalence of PTSD (Neuner et al. 2004; Wilker et al. 2015). Therefore, stronger and more complex neurobiological changes are to be expected after experiencing different traumas (Elbert and Schauer 2014).

A major limitation of this study is that we only measured healthy participants. Moreover, the experimental trauma includes features of witnessing a trauma only. Patient samples with PTSD may reveal additional effects and stronger neural changes during resting state immediately after trauma exposure. In addition, a replication of our study using different types of experimental trauma should be made. It should also be considered that both functional as well as structural neural correlates might differ already before traumatization (vulnerability factor) and this may lead to altered processing of a traumatic event. Future studies should focus more on predefined vulnerability factors such as hippocampal atrophy (Gilbertson et al. 2002) and differences in encoding before traumatization, in order to further specify predictive factors and potential preventive treatments in PTSD. As our sample of healthy subjects only included females, this further limits our findings. Up to 50% of females develop a PTSD after sexual assault (Chivers-Wilson 2006). Nevertheless, as similar physiological, emotional, and memory effects of the trauma film paradigm were found in female and male subjects in previous research (Weidmann et al. 2009), we would expect a potential generalization of immediate changes in functional connectivity to male subjects. However, whether these immediate changes in functional connectivity affect exactly the same brain regions in males as in females needs to be clarified. Therefore, future research should focus on whether our precise results in females can be generalized to male subjects.

Nonetheless, our study gives new insights into functional connectivity changes due to experimental trauma exposure, and highlights the mechanistic nature of these changes in relation to typical trauma manifestations. With the reported alterations occurring immediately after experimental trauma, preventive and targeted medical treatment can be deployed. Future research should focus on interventions that might alter connectivity changes to diminish maladaptive effects on the brain both immediately after trauma exposure and during the following days and weeks.

## Electronic supplementary material

ESM 1 (DOCX 254 kb)
