# Supplementary material for: Experimental trauma rapidly modifies functional connectivity
Source: Brain Imaging Behav. 2020 Sep 28;15(4):2017–30. doi: 10.1007/s11682-020-00396-2 (PMC8413225; doi:10.1007/s11682-020-00396-2)
Supplement: Supplementary file 1 — (DOCX 254 kb) [file 11682_2020_396_MOESM1_ESM.docx]

**Supplementary Material**

**Experimental trauma rapidly modifies functional connectivity**

Geraldine Gvozdanovic^,1,2, 3*^, Erich Seifritz^1,4^, Philipp Stämpfli^1^, Antonietta Canna^5^, Björn Rasch^6^, Fabrizio Esposito^5^

1. Department of Psychiatry, Psychotherapy and Psychosomatics, Psychiatric Hospital, University of Zurich, Zurich Switzerland
2. Institute of Psychology, University of Zurich, Zurich, Switzerland
3. Zurich Center for Neuroeconomics, Department of Economics, University of Zurich, Zurich, Switzerland
4. Competence Center of Sleep & Health Zurich, University of Zurich, Switzerland
5. Department of Medicine, Surgery and Dentistry "Scuola Medica Salernitana", University of Salerno, Baronissi (Salerno), Italy
6. Department of Psychology, University of Fribourg, Fribourg, Switzerland

**Methods**


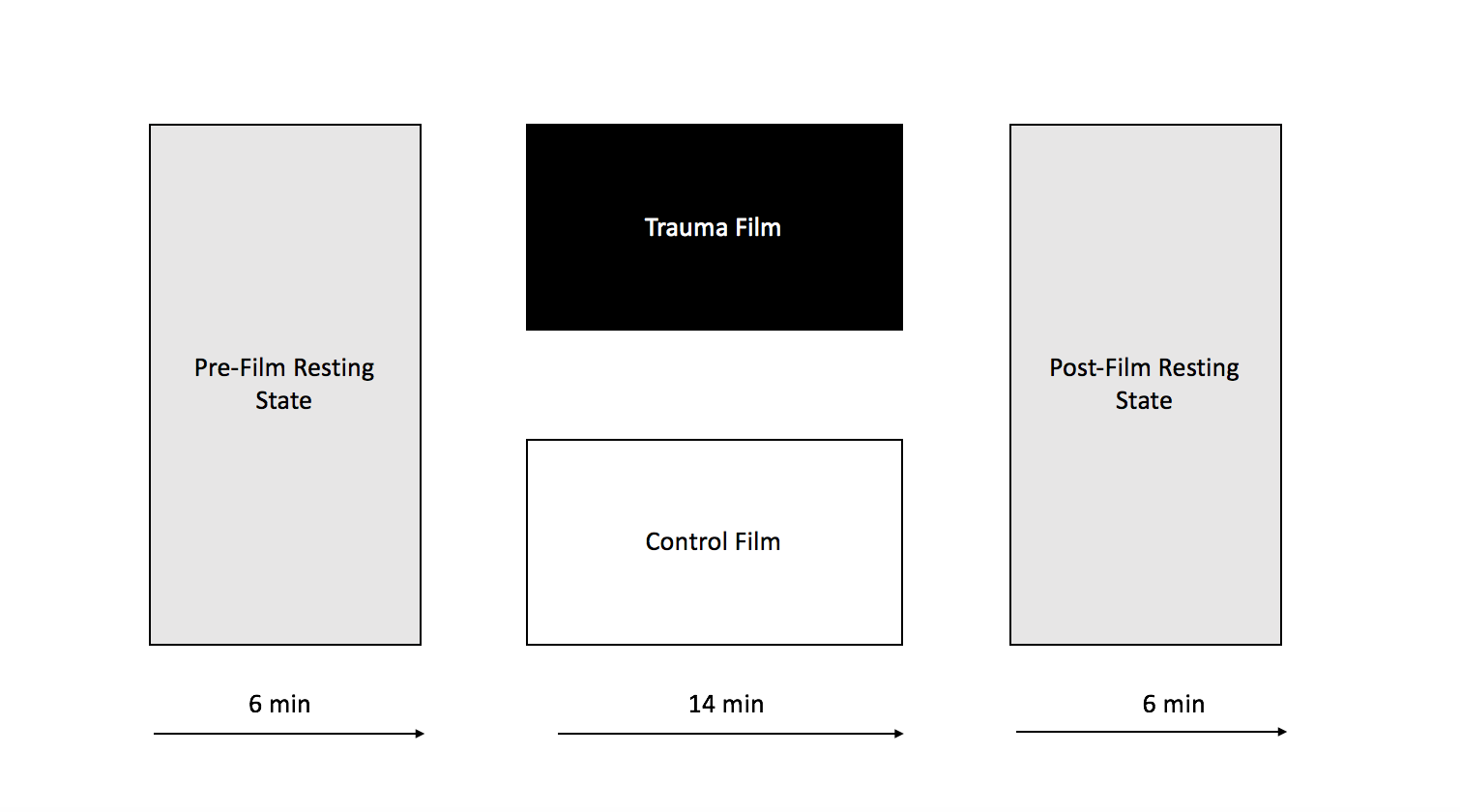


***Figure 1.*** *Procedure: Participants first had a resting state fMRI lasting 6 min. Participants were allocated to either a control film or a trauma film group, which was then again followed by another resting state phase of 6 min.*

**Results**

*Descriptive data: trauma diary (intrusions)*

| Sum of intrusions | Min intrusions | Max intrusions | Mean | Standard deviation |
| --- | --- | --- | --- | --- |
| Total number of intrusions | .0 | 13.0 | 3.87 | 3.22 |
| Intrusions Day 1 | .00 | 3.00 | 1.08 | .83 |
| Intrusions Day 2 | .00 | 4.00 | .92 | 1.04 |
| Intrusions Day 3 | .00 | 4.00 | .51 | .84 |
| Intrusions Day 4 | .00 | 3.00 | .49 | .73 |
| Intrusions Day 5 | .00 | 3.00 | .32 | .71 |
| Intrusions Day 6 | .00 | 3.00 | .32 | .71 |
| Intrusions Day 7 | .00 | 2.00 | .22 | .53 |

*Descriptive data: Group comparison of intrusions*

|  | Trauma film group  M ± SEM | Control film group  M ± SEM |
| --- | --- | --- |
| Total number of intrusions during 7 days | 3.87 ± 0.53 | 0.94 ± 0.23 |

*Descriptive data: emotional picture ratings*

| Control film group rating | Mean | Standard deviation |
| --- | --- | --- |
| Negative IAPS valence | 7.74 | 1.05 |
| Negative IAPS arousal | 7.31 | 1.34 |
| Neutral IAPS valence | .62 | .81 |
| Neutral IAPS arousal | .75 | .88 |
| Scrambled pictures valence | .64 | 1.16 |
| Scrambled pictures arousal | 1.21 | 1.79 |
| Film pictures valence | 1.39 | 1.52 |
| Film pictures arousal | 1.70 | 1.68 |

| Trauma film group rating | Mean | Standard deviation |
| --- | --- | --- |
| Negative IAPS valence | 7.32 | 1.49 |
| Negative IAPS arousal | 6.68 | 1.56 |
| Neutral IAPS valence | .52 | .63 |
| Neutral IAPS arousal | .62 | .81 |
| Scrambled pictures valence | .51 | 1.36 |
| Scrambled pictures arousal | 1.41 | 2.53 |
| Film pictures valence | 3.49 | 2.09 |
| Film pictures arousal | 3.94 | 2.05 |

*Brain behavior correlates:*

*Linear regression analysis*

|  | Regression coefficient (not standardized) | Standard error  (not standardized) | Standardized coefficients beta | t | sig |
| --- | --- | --- | --- | --- | --- |
| Hippocampus - Precuneus Connectivity | -.200 | .251 | -.134 | -.795 | .4 |
| Amygdala - MTG Connectivity | -.551 | .245 | -.380 | -2.249 | .03 |

Dependent variable: valence ratings of film pictures (delta film – scrambled)

*Amygdala seeds: ANOVA Results*

A detailed description on the statistical procedures of our fMRI data can be found in the *Image analysis* section of the manuscript. Analyses procedures are consistent throughout the entire paper.

We have found no significant main effect for neither film type (trauma film or control film) nor scan (pre- or post-film resting state) for the left amygdala. However, we have found a significant film type x scan interaction effect between the left amygdala and the right superior temporal gyrus (Fpeak = 17.23; Talairach [x y z] = [63, -1, -2]; cluster size = 465 mm3), transverse temporal gyrus (Fpeak = 21.67; Talairach [x y z] = [51, -22, 10]; cluster size = 2786 mm3), right sub-lobar (Fpeak = 18.25; Talairach [x y z] = [36, -13, 7]; cluster size = 362 mm3), right sub-gyral region of the frontal lobe (Fpeak =22.78; Talairach [x y z] = [12, -19, 58]; cluster size = 557 mm3) as well as the left postcentral gyrus (Fpeak = 22.58; Talairach [x y z] = [-15, -52, 67]; cluster size = 715 mm3), the left inferior frontal gyrus (Fpeak =17.40; Talairach [x y z] = [-39, 20, -2]; cluster size = 440 mm3) and the left superior temporal gyrus Fpeak = 21.00; Talairach [x y z] = [-54, -19, -2]; cluster size = 3226 mm3, extending to the middle temporal gyrus.

For the right amygdala, we found a significant main effect of film type in the left sub-gyral region of the temporal lobe (Fpeak = 16.99; Talairach [x y z] = [-30, 2, -38]; cluster size = 329 mm3). Moreover, there was a significant film type x scan interaction effect in the right superior temporal gyrus (Fpeak = 19.74; Talairach [x y z] = [63, 5, -2]; cluster size = 798 mm3), the right (Fpeak =17.50; Talairach [x y z] = [15, -19, 55]; cluster size = 350 mm3) and left (Fpeak = 20.50; Talairach [x y z] = [-12, -16, 49]; cluster size = 362 mm3) medial frontal gyrus, as well as the left middle temporal gyrus (Fpeak = 18.70; Talairach [x y z] = [-54, -19, -5]; cluster size = 404 mm3).

*Hippocampus/parahippocampal gyrus seeds: ANOVA Results*

There was a significant main effect of scan between the right hippocampus and the left precuneus (Fpeak = 20.11; Talairach [x y z] = [-3, -64, 37]; cluster size = 1348 mm3). There was no significant main effect for film type and no significant film type x scan interaction effect.

For the left hippocampus, there was a significant main effect for film type in the medial frontal gyrus (Fpeak =20.80; Talairach [x y z] = [3, 56, -8]; cluster size = 977 mm3). There was no significant main effect for scan and no significant film type x scan interaction effect.

*Head motion parameters*

To rule out the possibility that the observed effects were spurious effects mediated by head motion, we fitted a number of ANOVA models on our motion estimates to assess the significance of any factor or variable in terms of the influence of motion. The root mean squares of motion parameters were calculated per scan and individual. There were no significant differences in head motion parameters between the film groups (*p* = 0.83), between pre-film and post-film resting state scans (*p* = 0.69) and there was no significant motion by film group interaction effects (*p* = 0.88). Moreover, ratings on current mental states did not have any influence on motion parameters (for pre-film mental state: *p* = 0.40, for post-film mental state *p* = 0.52).

Mean FD did not significantly differ between the film groups (trauma vs control) in each scan and also did not significantly correlate with ratings on current mental state (all p > 0.1).

In sum, no effect of motion was found.
